# GnpIS: an information system to integrate genetic and genomic data from plants and fungi

**DOI:** 10.1093/database/bat058

**Published:** 2013-08-19

**Authors:** Delphine Steinbach, Michael Alaux, Joelle Amselem, Nathalie Choisne, Sophie Durand, Raphaël Flores, Aminah-Olivia Keliet, Erik Kimmel, Nicolas Lapalu, Isabelle Luyten, Célia Michotey, Nacer Mohellibi, Cyril Pommier, Sébastien Reboux, Dorothée Valdenaire, Daphné Verdelet, Hadi Quesneville

**Affiliations:** INRA, UR1164 URGI - Research Unit in Genomics-Info, INRA de Versailles, Route de Saint-Cyr, Versailles, 78026, France

## Abstract

Data integration is a key challenge for modern bioinformatics. It aims to provide biologists with tools to explore relevant data produced by different studies. Large-scale international projects can generate lots of heterogeneous and unrelated data. The challenge is to integrate this information with other publicly available data. Nucleotide sequencing throughput has been improved with new technologies; this increases the need for powerful information systems able to store, manage and explore data. GnpIS is a multispecies integrative information system dedicated to plant and fungi pests. It bridges genetic and genomic data, allowing researchers access to both genetic information (e.g. genetic maps, quantitative trait loci, markers, single nucleotide polymorphisms, germplasms and genotypes) and genomic data (e.g. genomic sequences, physical maps, genome annotation and expression data) for species of agronomical interest. GnpIS is used by both large international projects and plant science departments at the French National Institute for Agricultural Research. Here, we illustrate its use.

**Database URL**: http://urgi.versailles.inra.fr/gnpis

## Introduction

A second green revolution is needed to meet the challenges of the 21st century. Crop production of sufficient quantity is required (for both food and biofuel) while taking into account environmental consequences and climatic changes. Plant genetics and genomics have progressed, and there are now powerful tools for investigating the molecular basis of phenotypic variation, accelerating breeding and the exploitation of genetic diversity. Large volumes of crop genetic and genomic data have been integrated. However, these data have been produced by many different groups, and thus are heterogeneous and may even appear unrelated. The challenge for an efficient information system (IS) is to integrate these data by identifying their relationships both within the database and to publicly available material. The throughput of nucleotide sequencing technologies is becoming ever faster, and there is increasing need for powerful ISs, which are able to store, manage and explore large-scale data such that it can be fully exploited in the fields of genomics and genetics.

Several ISs have been developed. Some are dedicated to one species: TAIR (1) for *Arabidopsis*, MaizeGDB (2) for maize and Flybase (3, 4) for *Drosophila*. Others, such as Gramene (5, 6), Ensembl (7, 8), NCBI genome (9), Phytozome (10), PlantGDB (11), MIPSPlantDB (12) and Tropgene-DB (13), incorporate information for several species. Few databases offer an integrated multispecies system where navigation offers intuitive user interfaces for data browsing. By reducing the number of windows and databases required for a search, data exploration may be improved by limiting the risk of user confusion. Improved integrity of links used in the system should also increase the consistency of data. However, for data to be integrated according to researcher needs, it must be oriented towards specific fields of interest.

GnpIS was built to fulfil the needs of researchers for crop improvement. GnpIS stands for Genoplante Information System, derived from the name of a federative programme for plant genomics research in France, Genoplante (http://www.genoplante.com), which initiated its development. It initially integrated expressed sequence tag (EST) data for agronomical gene targets (14) and has then been extended to include genetic, genomic and transcriptomic data. GnpIS in its current form is the result of a decade of data integration from many other scientific programmes, user interactions and recurrent development. Several plant science departments at the French National Institute for Agricultural Research (INRA) and many large international collaborative projects use this system to manage and explore their data. We present some of these projects to illustrate the use of GnpIS in this article.

## Materials and Methods

### GnpIS architecture

**Figure 1.**
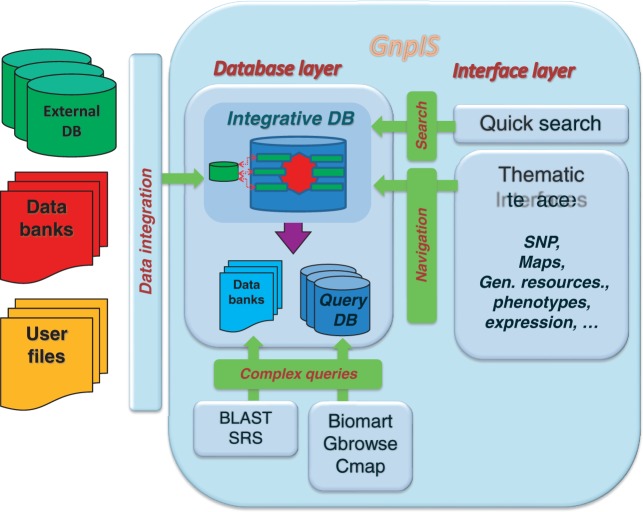
GnpIS architecture overview.

GnpIS is a modular web-based information system relying on cutting edge databases and data warehouse technologies (see ‘GnpIS software technologies’ in Supplementary information). It contains an ‘integrative database’ (composed by database modules), ‘query databases’ (also called ‘datamarts’) and ‘databanks’ (Figure 1).

**Figure 2.**
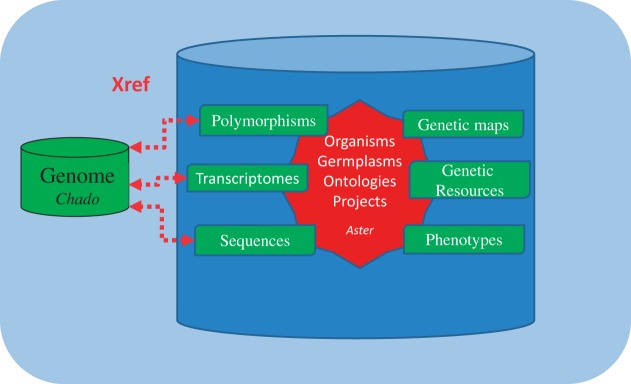
GnpIS integrative schema.

The ‘integrative database’, physically implemented in a single database schema, is conceptually organized around several modules, which gather tables related to scientific fields. A core module, called ‘Aster’ (Figure 2), contains transversal tables shared between several scientific modules like organisms, germplasms, ontologies and projects. The scientific modules handle various types of data: sequences, genetic markers and maps, genetic collections, phenotypes, transcriptomes and DNA polymorphisms. Key scientific entries like germplasm, organism, ontology, and project serve as pivots for interoperability. Key attributes linking data in the IS are present in these tables. Taxon, identifiers for accession (or lot), respectively, from table organisms and germplasms allows to link data available for a given species and genetic resources. A project identifier in the project table links data produced in the same scientific project, allowing retrieving data consistently produced to answer specific biological questions. Ontologies are stored in a central table for consistency between other modules, also allowing to link data described by the same ontology term. This schema reduces data redundancy in the database, improves data consistency and increases query performances by reducing the number of SQL joins. This single integrative system is accessed through module-oriented interfaces (also called ‘thematic interfaces’) and is queried from a unique portal. It is the key structure that allows GnpIS to bridge genetic and genomic data.

Dedicated modules store data specific to a scientific field. The ‘sequence module’ stores EST and mRNA sequences, which are produced by Sanger sequencing and by new sequencing technologies (Solexa, 454, HiSeq2000). Short reads produced for single nucleotide polymorphisms (SNP) identification and RNA-Seq analysis can be also stored here. The ‘genetic map module’ is dedicated to genetic maps, markers, traits, quantitative trait loci (QTLs) and QTL meta-analysis. Interoperability between these two modules allows users to aggregate data and find co-localizations between genes (if their EST or mRNA is genetic markers) and QTLs. There are also bidirectional links with markers that have been mapped on the genome as annotations (see further ‘genome module’). The ‘transcriptome module’ stores raw and normalized data and gene lists resulting from diverse expression experiments, such as microarray (with cDNA and probes) and macroarrays (with high density filters). Exploiting the links between ‘transcriptome’ and ‘genome modules’, the user can find biological information, or annotation, relating to gene expression. The ‘polymorphism module’ stores SNP, small insertions and deletions between genetic resources accessions. Links are established with the ‘genetic resources module’ to provide details about the accession, and to the ‘genome module’ to retrieve the sequence variations in their genome annotation context. This module is able to store large-scale data such as SNPs found in next-generation sequencing (NGS) experiments. The ‘genome module’ is based on the ‘Chado’ database model (15) and cross-references with other GnpIS modules. It contains genomic sequence data, structural or functional annotations and a synthetic view of all GnpIS data that can be mapped on a genome. For data management efficiency, GnpIS provides one ‘genome module’ per species. This module is also able to store information on the syntenic relationships between closely or distantly related species as well as physical maps. The ‘genetic resources module’ manages passport descriptors (accession/germplasm identification, taxonomy, geographical origin, pedigree) and phenotypes for plant collections. It is linked to the polymorphism module through recorded germplasm names. The ‘phenotype module’ is dedicated to the storage of phenotype data produced during genotype-environment (climate, soil) interaction studies. Phenotypes can be described with ontologies and linked to data with ‘genetic resources’ and ‘polymorphisms modules’.

### Data management

GnpIS is built using well-defined processes and procedures for efficient data management. This helps assure the robustness and quality of the system.

Data are uploaded using a dedicated submission web portal using standards when available (e.g. FASTA, GFF3, VCF), output files from the main software of the field or as tabulated text files or spreadsheets. The submission web portal describes accepted files and data formats. Files not only can be manually provided but also be automatically generated from other databases using extract transform load software or scripts. Deposited data files are automatically checked when deposited on the web to ensure a proper data submission. Accepted data types are annotations, gene expression, DNA polymorphism, genetic markers, genetic and physical maps, genetic collections and phenotypes.

Data access is controlled through user accounts. A hierarchy of roles (select/update/insert/delete/admin) is granted according to user access rights. At the database level, fine-grained confidentiality level is maintained using table fields, which have values assigned according to corresponding user groups. ‘SQL views’ are used to restrict access according to users’ groups. Thus, data can be kept confidential and restricted to a particular group of users before publication.

Data release policy endorses the open data model. Data are made public as soon and as much as possible. However, data confidentiality can be maintained before the end of a project or before the publication of the scientific article describing the work to preserve authors’ intellectual properties.

### Queries, data searches and navigation

Users can query GnpIS using (i) a quick search mode, (ii) an advanced search mode and (iii) thematic module-oriented navigation.

The quick search mode uses Lucene, a high-performance full-featured text search engine library (http://lucene.apache.org). Users enter words (or part of words) in a text box as with the ‘Google search engine’. Matching words are returned to the user with details of the text hit and a link to the corresponding data. This mode is useful when users are unaware of the location of the data in the repository.

Advanced search interfaces are used to combine search criteria. The ‘integrative database’ schema is optimized for data integration, but is not efficient for data retrieval in response to complex queries. To improve the performance of complex queries, ‘query-databases’ (‘datamarts’) are built by extracting and reorganizing the data in another schema tuned for specific queries. The GnpIS includes sets of ‘datamarts’ built to answer specific questions according to current user needs. For example, ‘datamarts’ may be focused on genome annotation or be orientated towards genetic markers or SNPs. This ‘advanced search tool’ is based on BioMart (16), a query-oriented data management system allowing the rapid development of such datamart and query-builder interfaces. The system incorporates data-mining features and can be used for searches of complex descriptive data. Converting a data source into a BioMart data set is fully automated by the tools included in the package. BioMart allows efficient filter-based queries to be built: for example, queries to find all SNPs mapped at a gene of interest or to retrieve all QTLs found for a species in a specific genomic region. Results contain web links to appropriate GnpIS data such that further details and interpretation can be found. Results can be exported in various flat formats for analysis, or uploaded into the Galaxy tool (17, 18), to be used in workflows (see later in the text).

Other tools are also used in another type of ‘query database’. GBrowse2 (19) instances dedicated to each species are used to browse genome annotations stored in the Bio::DB::SeqFeature::Store (MySQL) data model, with NGS data in BAM files. GBrowse_syn (19, 20) is used to display genome synteny. CMap (21) is fed with genetic data extracted from GnpIS to serve those scientific communities or users who prefer to display genetic maps with this tool.

Users can navigate through the data using either a quick search query, an advanced search with BioMart or a simple query performed directly from a thematic web interface. These interfaces have been developed using JSP technology or other web-dedicated languages such as CSS and JavaScript. Search results exhibit links to more specific areas of information, narrowing the search for the desired data.

The sequence retrieval system (22) is used to query and extract DNA or protein sequences from databanks. BLAST (23, 24) and Blat (25) servers, either from sequence retrieval system or Mobyle (26), allow searches by similarity to sequences in the databank.

Search and retrieval performance are direct consequences of both database schema properties and stored data. Compared with other similar plant databases, which focus generally on genomics, genetic data are here the primary focus of our integration schema. Genetic maps, genetic markers (RFLP, SSR, SNPs, etc.), QTL, phenotypes and accession identifiers are in the heart of the system allowing storing and integrating them in details. Other databases also focus on these data, but they are stored in independent databases limiting data consistency and the possibilities of integration across the whole genetic field. Datamarts provided under their respective Biomarts instances reflect these differences. Although some provide only genomics datamarts or poorly integrated datamarts across different databases, GnpIS provides highly integrated genetic data.

## Results

### An efficient data querying and browsing system

Ergonomic navigation is continuously improved by user interactions and recurrent system amendments. The international scientific community can access GnpIS through a single entry point with intuitive interfaces and search tools (http://urgi.versailles.inra.fr/gnpis). Users can access data using quick search or advanced search tools (as described in ‘Materials and Methods’ section) or focus their search on a specific topic such as (i) genetic maps and QTLs, (ii) structural and functional gene annotation, (iii) sequence polymorphisms, (iv) phenotyping or (v) gene expression. Dedicated query interfaces (‘thematic interfaces’) are available. Users can obtain lists of results or data cards that compile all information describing the requested object with links to data stored in the system or remote databases.

For example, from the genetic data thematic interface, users can be guided to find QTLs from (i) selected maps, (ii) selected traits or (iii) by name. Results are provided as a list of QTLs or cards describing, for example, a marker or genetic map. Users can explore the QTL genomic context further by following web hyperlinks to related feature in GBrowse. For species that have an annotated genome, it is possible to access structural and functional annotation of genes in the genomic region corresponding to the QTL. Users can also make SNP-focused queries, searching for SNPs found in a set of genotypes or lines associated with a location on a genome. SNPs can also be retrieved according to their genomic location. The results provided to users will be, for example, (i) the gene and the line in which the SNP was found, (ii) links on the current SNP card and on the line card or (iii) a link on the accession card (germplasm) (if referenced in the ‘genetic resources module’) that will give the corresponding passport descriptors and measured phenotypes.

A quick search can be performed with words or combinations of words used to name or describe QTLs, markers, genes, gene annotation, projects and so forth. The GnpIS quick search tool displays results in different tabs according to the nature of the information. The user can identify matches of interest by choosing the tab in the list displayed. List of results is sorted by relevance, according to the text match coverage in the indexed text field. Moving the mouse over a result will display a pop-up where additional information can be found. The hyperlink available on each result allows data access using the appropriate interface. Users may find the requested information rapidly or refine the query if needed by changing the searched term or entering string search operators in the search text box.

BioMart-based advanced search can receive (i) all QTLs mapped on a genome sequence and associated mapping information, (ii) all genetic markers and predicted genes that are located in a genomic region [specified by its coordinates in base pair (bp)] with details on maps, markers and genetic position in centimorgan (cM) and (iii) all SNPs for a gene or a set of genes with details of their 5′ and 3′ flanking sequences and variation pattern. BioMart has its own website that can be configured and customized. Access to data sets is provided through dedicated query forms adapted for scientific questions. Programmatically, it can be accessed through web services or API written in Perl and Java. BioMart can cross-query two data sets, even by remote connecting to sites over the Web. It may, therefore, be considered as a meta-database management system that integrates multiple dispersed database systems over a computer network. Through data abstraction, this federated database system can provide a uniform user interface and enable users to manipulate data in several dispersed databases using a single operation. A user may query our system, cross the results with external BioMart data sets remotely and access it through BioMart built-in web services.

Users may refine their analysis using the URGI Galaxy system [a local instantiation of Galaxy (17)]; this software system provides a simple web-based interface for a variety of tools and, through them, access to databases. Galaxy is able to connect to GnpIS, BioMart and other distant BioMart sites to query and retrieve data. Tools may be chained in workflows possibly shared by users. This allows users without skills in informatics or programming to perform complex large-scale analysis using just a web browser. Manipulation of data in Galaxy is easy; many operations are possible on spreadsheets. These can be joined according to column values and manipulated in a similar way to tables in databases. Galaxy can also integrate heterogeneous data from several different databases and user files (18).

### A data repository used by international consortia for large-scale projects

GnpIS is a multispecies information system, which can integrate large-scale data. Its infrastructure has efficient and reliable storage capacities, ensuring data consistency and long-term conservation of information.

GnpIS is used to manage, store and display data collected during large collaborative projects. Data from INRA and other international projects have been collated for >10 years. They contributed and benefited from system development. The French Genoplante initiative chose GnpIS to store its data, making available in the system, data from many species (see Table 1 and ‘Data summaries’ in Supplementary Data).

**Table 1.** GnpIS data summary

Data types	Taxons	Experiments	Features
Genetic maps	7		68
Genetic markers	7		32 896
QTL	2	32	819
MetaQTL	1	11	19
SNP	42	449	193 519
Indels	42	197	10 441
Expressions	5	8	103
Genome	8		11
Genes	8		818 867
Genetic resources	4772		16 587
Phenotypes	4772		80 768
Phenotypes (GxE)	6	3	131

The number of species represented and the demand for data integration are growing. GnpIS is currently used for two plant pathogen fungi, *Leptospheria maculans* (27) and *Botrytis cinerea* (28). Gene and repeat annotations are displayed with the genomic sequences. The Gene Report System, connected to the ‘genome module’, provides detailed annotation on gene structure and reference to the relevant information sources [e.g. Blast (23, 24) and Interproscan (29) results or peptide signal predictions]. Transcriptome data are available for *B. cinerea*. Genome synteny is shown (i) between two strains of *B. cinerea* and the closed related species *Sclerotinia sclerotiorum* (ii) between seven *Leptosphaeria* species.

Tree data are also stored in the GnpIS. Poplar and apple tree genome sequences, and their annotations, are displayed alongside SNPs, genetic markers and QTLs. With the development of NGS, large numbers of SNPs in tree species (e.g. oak, maritime pine) are being stored without a reference genome sequence.

Recently, work has focused on two crops: grapevine and wheat. The INRA has been working on grapevines for many years. Genetic and genomic programs have generated huge amounts of data, which are now integrated in GnpIS; this includes genetic maps, markers and QTLs. The International Grapevine Genome Program (IGGP) chose GnpIS to manage their genomic sequence data and annotations, for example, for the *Vitis vinifera* 12X assembly sequence (30, 31). Transcriptomic data from microarrays, and SNP data from large national and European projects, have been integrated. Thus, data can be explored in their genomic context, and the system is able to offer scientists an integrated view of the grapevine genome, its annotations, expression and polymorphisms.

**Figure 3.**
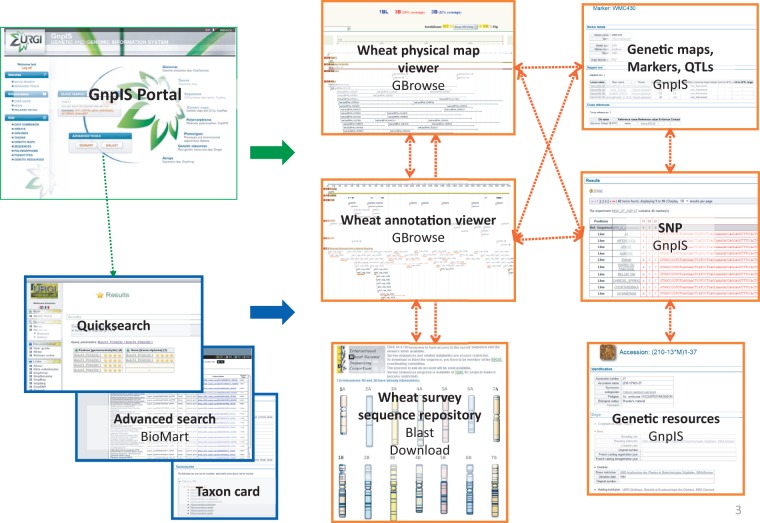
GnpIS wheat navigation.

INRA has also accumulated large volumes of data for genetic improvement of wheat. GnpIS stores these data, offering an integrated view to the wheat research community (Figure 3). Bread wheat genetic maps, markers and QTL are available. GBrowse2 with the MySQL Bio::DB::GFF schema (32) is used to manage physical maps in GnpIS. Contextual pop-up menus provide links to the genetic markers and sequences produced. Researchers can navigate through genetic maps, physical maps and the already available sequences of this genome. Available annotations are displayed on the sequences as well as the polymorphisms, genetic markers and QTLs. The International Wheat Genome Sequencing Consortium (IWGSC) uses GnpIS as a repository to manage and display wheat sequences to the scientific community.

## Discussion

### Data interoperability and integration

In computer science, interoperability refers to the capability of diverse software to work together by exchanging data. This is made possible by the use of a common set of exchange formats and of compatible protocols. Software able to read and write the same file format may have basic level of interoperability, but web services require more elaborated forms. In databases, a simple level of interoperability can be achieved through cross-reference linkages.

Data integration uses data interoperability to combine data from different sources. Integration provides users with a unified view of these data. Two architectures are able to support this concept: virtual databases (also known as federated database systems) and data warehouses.

A federated database is a meta-database management system that transparently integrates multiple dispersed database systems over a computer network. Federated database systems use data abstraction to provide a uniform user interface and enable manipulation of data in several dispersed databases via a single operation. To achieve this, the system breaks down the initial query into sub- queries for submission to the individual databases. The set of results returned is then composed into a single result. Federated database systems use wrappers to translate languages that may differ between database management systems.

A data warehouse aggregates data for reporting, whereas a production database aims to store data efficiently. A data warehouse will load aggregated data taken from one or several production databases. The raw data are cleaned, transformed, catalogued and made available for navigation and data mining. Architecturally, a data warehouse provides a high level of data consistency, as the data reside together in a single repository at query time (unlike in federated databases). It also ensures referential integrity: referential integrity is the property that a record cannot be deleted if it contains a value referred to by another record. Some relational database management systems enforce referential integrity by either deleting the referencing record or returning an error when deleting. Data warehouses are more difficult to maintain and to update than Federated databases, but offer better data consistency. In practice, data warehouses tend to be associated with datamarts (databases obtained by extracting a data subset, organizing and storing according to user needs). A datamart design is often based on an analysis of user needs, and hence emphasizes ease of access and usability for a particular purpose. In contrast, a data warehouse design may be based on an analysis of exisiting data and how it can be stored for later use.

The GnpIS architecture is based on a data warehouse approach to ensure the best possible data consistency. We associated datamarts so that the system could be adapted to user needs. The main advantage of this structure is that data access can be adapted without requiring modification of the data warehouse schema. Modifying such a database is complex, as the normalized schema may involve hundreds of tables and a large amount of data would need to be transformed to fit the new normalized schema. The adapted datamart schema improves end-user response time by reducing the number of joins required to fulfil a query. Datamart schemas are not normalized and require more storage space but, as searches are focused on a specific topic, only a subset of the data is needed. Fewer tables and fields are required, allowing datamarts to be implemented at a lower cost than data warehouses. When the underlying data in the reference database change, the existing data in the datamart are not transformed, but are instead replaced by new data extracted from the reference database. Datamarts also help to define group of users, facilitating the potential to define their specific needs further. GnpIS also uses BioMart to facilitate database federation, making our system interoperable through other BioMart or Galaxy systems without the need of developing web services (Biomart also provide them).

### A collaborative system

GnpIS is the result of more than a decade of data integration, recurrent development and user interactions. It has been developed alongside scientific projects, involving researchers in plant genomics or bioinformatics. It helps scientists to manage and explore their data, using interface and database designs guided by their needs. The close collaboration between researchers, engineers, biologists and bioinformaticians contributing to the collaborative projects has helped to drive its development. Working groups have been established and agile software development methodologies have been used to capture user needs, contributing to the success of the system. Short iterative development processes improve the reactivity to changes, as frequent releases permit users to give rapid feedback. Software evolution is thereby closely guided by end users. Meetings, software demos, training sessions and video conferences are organized to maintain the communication between collaborators and ensure a useful evolution of the software. A dedicated web page ensures this communication (http://urgi.versailles.inra.fr/Platform/Training)

Fifty-six scientific projects have used GnpIS since its creation. These projects were realized either in the framework of collaborative projects from researchers from the plant science departments at the INRA or large international collaborative projects such as GrapeReSeq, TriticeaeGenome, IWGSC, IGGP (see detailed list at http://urgi.versailles.inra.fr/Projects/). Scientists use the system to not only explore and share their data but also coordinate their work. The benefit they gain from such a system is also visibility and funding. Hence, a number of scientific projects have been successfully funded because their expected results will be made available to the community through GnpIS. Scientists and funders consider that it is crucial to rapidly and widely release data to the scientific community in a way that they could be reused and compared with other available data. Several new projects were built from GnpIS data and have enriched in return the IS. The links between the TriticeaeGenome project (http://wheat-urgi.versailles.inra.fr/Projects/TriticeaeGenome2) and the wheat sequencing project conducted by the IWGSC is a perfect illustration. The Triticeae Genome project produced some of the physical maps used for sequencing the wheat genome. The wheat sequences are then inserted and linked to the physical maps showing their relationships.

## Conclusions

A major challenge in plant genomics is to find genes related to agronomical traits, such as those involved in yield and quality (e.g. wheat), in disease resistance (e.g. grapevine) or in stress adaptation (e.g. poplar). Easy navigation through, and the consolidation of, genetic and genomic data is required for work to create new crop varieties with improved nutritional and environment characteristics. GnpIS relies on state-of-art technologies and methodologies and has been shown to be a flexible and extendable system. It is an original information system that allows users to navigate easily through heterogeneous data, and simultaneously query different scientific domains. It stores data from many diverse studies in the same database. As a reference data warehouse, it can also be used to promote data exchange and dissemination to the whole scientific community. It can be used for various organisms of particular interest and which are the subjects of international collaboration, including wheat (IWGSC), grapevine (IGGP) and plant pathogen fungi (such as *L. maculans* and *B*. *cinerea*). GnpIS is able to manage data confidentiality within the public domain while also supporting data extraction for external analysis.

## Supplementary Material

Supplementary Data
