# Supplementary material for: GnpIS: an information system to integrate genetic and genomic data from plants and fungi
Source: Database (Oxford). 2013 Aug 19;2013:bat058. doi: 10.1093/database/bat058 (PMC3746681; doi:10.1093/database/bat058)
Supplement: Supplementary Data [file supp_2013_bat058_index.html]

Supplementary Data 

# GnpIS: an information system to integrate genetic and genomic data from plants and fungi

## Supplementary Data

files

**Files in this Data Supplement:**

- Supplementary Data - doc file
