# Supplementary material for: GnpIS: an information system to integrate genetic and genomic data from plants and fungi
Source: Database (Oxford). 2013 Aug 19;2013:bat058. doi: 10.1093/database/bat058 (PMC3746681; doi:10.1093/database/bat058)
Supplement: Supplementary Data [file supp_bat058_GnpIS2012-SuppInfV3final.doc]

# Supplementary information

## GnpIS software technologies

GnpIS is built using the Service-Oriented Architecture (SOA), an approach for constructing complex software-intensive systems from a set of universally inter-connected and interdependent building blocks (or services). The platform chosen for the implementation of the Service-Oriented Architecture is the J2EE platform (http://java.sun.com/javaee) from Sun Microsystems™ (http://www.sun.com). This software architecture relies on five layers: presentation, orchestration, services, domain and persistence. The presentation layer is the customer layer of the application, the web browser in the case of GnpIS. The orchestration layer facilitates communication between the customer and services using dedicated protocols such as HTTP or RMI. Services are the central layer of the application; they are distinct units that can be combined or used separately to provide software functionalities. The domain layer is the business layer of the application. It mainly contains query and data management functionalities. The persistence layer generally relies on a Relational Database Management System (RDBMS) and on tools, which allow interactions with it. We use Oracle™ (http://www.oracle.com) as our RDBMS but the use of an Object/Relational Mapping (ORM) tool, such as Hibernate, allows us to easily switch to another RDBMS (for example, PostgreSQL or MySQL). Hibernate facilitates mapping from Java classes to database tables and provides data query and retrieval facilities. The modularity of SOA architecture supports many opportunities for software development. The various components are partitioned to facilitate maintenance and development of the tools produced. For example, all database queries are located in the same layer. Developers can therefore specifically target source code to fix a bug or add a new feature. GnpIS uses the Model-Driven Architecture (MDA) approach for software design. It separates business and application logic from underlying platform technology. The model outlines the main technical specification for our system; the different layers and the way they interact. This platform-independent model documents the business functionality and behaviour of an application independent of the technology-specific code that implements it. The use of a code generation framework reduces code writing time for certain parts of our applications, particularly the services, domain and persistence layers of the SOA. The framework is used to perform the tedious and complex tasks needed to implement the business logic described in the model. The model must then respect some guidelines (such as naming constraints, stereotypes or tagged values) to finely tune our code generation framework. The automatic generation tool we use is AndroMDA (http://www.andromda.org). It can be extended, with our own custom designed components, called cartridges, which could be seen as plug-ins. This generation step creates many Java classes and XML files involved in the configuration of the two main external libraries that GnpIS modules use (Hibernate (https://www.hibernate.org) and Spring (http://www.springsource.org)). AndroMDA generates a clean mould for the domain and services layers and generates the full configuration for Hibernate. The logic of the components, such as queries or data transformations, need further implementation.

The orchestration layer of our application is based on the Model-View-Controller 2 (MVC 2) framework design. Here, the application flow is mediated by a central Controller. The Controller transfers requests - in our case, HTTP requests - to the appropriate handler. The handlers are tied to a Model, and each handler acts as an adapter between the request and the Model. The Model (equivalent to the Domain layer) represents, or encapsulates, an application's business logic or state. The response is usually then passed back through the Controller to the appropriate view. The Controller manages requests and responses using a set of mappings which are usually loaded from a database or configuration file.

We use Spring, a J2EE framework which implements many concepts and provides useful functionalities. The main Spring modules we have included in GnpIS are (i) the Core, (ii) the DAO, (iii) the ORM and (iv) the webs modules. The Core module is the most fundamental part of the framework, providing IoC (Inversion of Control) and DI (Dependency Injection). It facilitates sophisticated implementation of the Factory design pattern, removing the need for programmatic singleton classes and allowing us to decouple the configuration and specification of dependencies from our program logic. The DAO module provides a JDBC-abstraction layer that removes the need to do tedious JDBC coding and parsing of database-vendor specific error codes. It also allows straightforward management of transactions. The ORM module provides integration for Hibernate, our Object/Relational Mapping tool. The Web module provides integration for the web framework implementing MVC2.

Our last external tool is Struts (http://struts.apache.org). It is a web-application development framework implementing the Model-View-Controller 2 (MVC 2) design pattern. It provides three key components: (i) a request handler mapped to a standard Uniform Resource Identifier, (ii) a response handler that transfers control to another resource for response completion and (iii) a tag library that helps the developer to create interactive form-based applications with Java Server Pages (JSP).

## Data summaries

| Genetic map data | Taxons | Maps | Markers | QTLs |
| --- | --- | --- | --- | --- |
| Total | 7 | 68 | 52245 | 819 |
| Examples | *Triticum aestivum* | 26 | 32745 | 749 |
|  | *Zea mays L.* | 7 | 7936 | 0 |
|  | *Vitis vinifera L.* | 27 | 1284 | 0 |
|  | *Hordeum vulgare* | 3 | 7123 | 0 |
|  | *Brassica napus L.* | 2 | 383 | 70 |

| Polymorphism data | Taxons | Experiments | SNPs, insertions, deletions | Genotypes |
| --- | --- | --- | --- | --- |
| Total | 5 | 360 | 66345 | 672 |
| Examples | *Triticum aestivum* | 10819 | 10819 | 69 |
|  | *Arabidopsis thaliana* | 33552 | 33552 | 265 |
|  | *Brassica napus* | 2628 | 2628 | 45 |
|  | *Pinus pinaster* | 7357 | 7357 | 154 |

| Expression data | Taxons | Experiments | Hybridizations | Arrays | Gene Lists |
| --- | --- | --- | --- | --- | --- |
| Total | 5 | 4 | 68 | 56 | 12 (35690 genes) |
| Examples | *Botryotinia fuckeliana* | 1 | 8 | 8 | 3 |
|  | *Botryotinia fuckeliana in interaction with Arabidopsis thaliana* | 1 | 34 | 22 | 3 |
|  | *Botryotinia fuckeliana in interaction with Helianthus annuus* | 1 | 17 | 17 | 3 |
|  | \| *Sclerotinia sclerotorium*  *fuckeliana in interaction with Helianthus annuus* \|  \| \| --- \| --- \| | 1 | 9 | 9 | 3 |

| Plant genomes | Taxons | Genes | SNPs | Markers | QTLs |
| --- | --- | --- | --- | --- | --- |
|  | *Triticum aestivum (RPH7 region)* | 203 | 92 | 140 | 1 |
|  | *Poplar version 1* | 46830 | 0 | 3655 | 19 |
|  | *Vitis vinifera L.* | 254060 | 465501 | 487 | 0 |
|  | *Zea mays* | 359994 | 0 | 804 | 1150 |

| Fungi genomes | Taxons | Assembly size (Mb) | Scaffolds | Genes | Transposable elements |
| --- | --- | --- | --- | --- | --- |
|  | *Botrytis cinerea T4* | 39.5 | 118 | 16360 | 792 |
|  | *Botrytis cinerea B0510* | 42.3 | 588 | 16448 | Na |
|  | *Sclerotinia sclerotiorum* | 38.3 | 36 | 14522 | Na |
|  | *Leptosphaeria maculans* | 45.12 | 76 | 12469 | 6089 |

| Genetics resources | Taxons | Accessions | Phenotypes |
| --- | --- | --- | --- |
| Total | 4870 | 16391 | 80628 |
| Examples | *Vitis* | 4451 | 7862 |
|  | *Medicago* | 98 | 2457 |
|  | *Triticum (bread wheat)* | 32 | 2114 |
|  | *Forage and turf* | 29 | 537 |
|  | *Barley* | 9 | 461 |

| Phenotypes | Species | Trials | Accessions | Phenotypes |
| --- | --- | --- | --- | --- |
| Total | 3 | 3 | 198 | 63 |
| Examples | *Hordeum vulgare* | 1 | 9 | 13 |
|  | *- Prunus domestica L.*  *- Prunus insititia* | 1 | 66 | 32 |
|  | *- Vitis L.*  *- Vitis vinifera subsp vinifera cv. Gewürztraminer*  *- Vitis vinifera subsp vinifera cv. Riesling* | 1 | 123 | 18 |
